# Quenched hydrogen-deuterium exchange NMR of a disease-relevant Aβ(1-42) amyloid polymorph

**DOI:** 10.1371/journal.pone.0172862

**Published:** 2017-03-20

**Authors:** Marielle Aulikki Wälti, Julien Orts, Roland Riek

**Affiliations:** Laboratorium für Physikalische Chemie, ETH Zürich, Zürich, Switzerland; University of Akron, UNITED STATES

## Abstract

Alzheimer’s disease is associated with the aggregation into amyloid fibrils of Aβ(1–42) and Aβ(1–40) peptides. Interestingly, these fibrils often do not obtain one single structure but rather show different morphologies, so-called polymorphs. Here, we compare quenched hydrogen-deuterium (H/D) exchange of a disease-relevant Aβ(1–42) fibril for which the 3D structure has been determined by solid-state NMR with H/D exchange previously determined on another structural polymorph. This comparison reveals secondary structural differences between the two polymorphs suggesting that the two polymorphisms can be classified as segmental polymorphs.

## Introduction

Alzheimer’s disease is the most fatal neurodegenerative disease characterized by the deposition of intracellular neurofibrillary tangles and extracellular plaques in the brain. These plaques consist mainly of aggregated Aβ(1–42) and Aβ(1–40) peptide, forming amyloid fibrils of different kinds called polymorphisms [1, 2]. Polymorphism in disease-associated amyloids are a phenomenon [3–8], in which a given peptide or protein sequence adopts two or more structurally distinct amyloid conformations under the same environmental condition. At the mesoscopic level under the electron microscope for instance, amyloid polymorphs may be detected by their distinct fibril morphologies such as different degrees of twist, number of protofilaments per fibril, or their diameters or mass per unit fibril length as documented for *in vitro* prepared Aβ amyloid fibrils for example [9]. At the atomic level, polymorphisms are detected by multiple NMR signals per atom in solid-state NMR spectra of various amyloids including Aβ(1–40) and Aβ(1–42) [10, 11]. In addition, crystal structures of amyloid peptides having the steric zipper cross-β-sheet motifs show different structures as exemplified for example for the peptide segments of Aβ(1–42) 16-KLVFAA-21 and 35-MVGGVVIA-42 [5]. To reveal structural differences at the secondary structure level quenched hydrogen/deuterium (H/D) exchange by NMR spectroscopy may be used since a slow exchange indicates protection of the amide from the solvent or/and involvement in a hydrogen bond [12, 13]. The latter method was used here to reveal secondary structural differences between a disease-relevant Aβ(1–42) amyloid fibril polymorph for which the 3D solid-state NMR structure was determined recently [10, 11, 14, 15] and a Aβ(1–42) amyloid fibril polymorph studied by us earlier for which H/D exchange data has been published [16].

## Results

### Sample preparation of a single polymorph

We recently determined the 3D structure of a disease-relevant polymorph of Aβ(1–42) amyloid fibrils by solid-state NMR [14]. For the structure determination it was important to have only a single polymorph, which was obtained by the following procedure: 30 μM of Aβ(1–42) was incubated in 100 mM Phosphate buffer, at a pH of 7.4, 100 mM sodium chloride (NaCl), and 100 μM zinc chloride (ZnCl). Three generations of seeding, each time taking 10% of the parent sample for the next generation, was preformed to obtain a homogeneous sample as determined by a single set of cross peaks in the solid-state NMR spectra [17].

### Screening of solvent conditions for dissolving the Aβ(1–42) fibrils in an aprotic solvent

For the NMR-based quenched H/D exchange measurements of the fibrils an aprotic solution must be found that dissolves the fibrils into solution-state detectable monomers. The usage of DMSO and small amounts of trifluoroacetic acid (TFA) has previously been successfully used for the dissolution of Aβ(1–42) fibrils [16]. In this study also 5% of D_2_O was added to minimize the effect of any residual water. Further, to keep the intrinsic exchange at a minimum, the pH of the sample was controlled by addition of acids (here TFA or dichloroacetic acid (DCA)). In a first step the dissolution of the fibrils had to be ensured. The fibrils were dissolved in DMSO, 5% D_2_O, and TFA at different concentrations, starting without TFA and going up to 0.1% (0%, 0.02%, 0.05%, 0.1%) or 2–5% DCA. The amount of TFA and DCA were chosen in a range where the intrinsic exchange rate should be minimal [12]. The dissolution of the fibrils was tested by SDS-page and by electron microscopy. In H_2_O and in DMSO, the fibrils were not dissolved properly as a lot of fibrils were found on the carbon grid by electron microscopy (S1 Fig). With the addition of increasing amounts of TFA less fibrils were observed and also more disordered-like aggregates were seen on the grids, indicating overall that some fibrils were dissolved (S1D Fig, data only shown for 0.1% TFA). It is noted however, that residual fibrils were found in all the samples by scanning carefully through the whole carbon grid (S1E Fig) albeit the amount of fibrils was qualitatively reduced compared to the samples dissolved in water or in DMSO only.

Next, the dissolution kinetics of the fibrils in the aprotic solvent were followed by measuring time-dependently the intensities of the peaks in the non exchanging methyl regions of the liquid state ^1^H 1D NMR spectra. Upon dissolving the fibrils in DMSO, 5% D_2_O, and 0.1% TFA the methyl intensity was increasing over time, indicating that remaining fibrils were dissolving over time. By decreasing the amount of TFA to 0.02% this process was interfered with and a stable sample was stabilized. Unfortunately, this result was not highly reproducible, which we attributed to the notion that such low concentrations of TFA are difficult to be controlled (i.e. errors in the dilution series). Therefore, we decided to switch to the acid DCA. DCA is less acidic than TFA and therefore higher amounts are required to achieve the same acidity, which makes the handling more accurate. EM pictures of fibrils dissolved in the solvent DMSO, 5% D_2_O, and 2% DCA looked similar to the ones in 0.1% TFA (S1 Fig). Even though the majority of the regions are crowded with disordered-like aggregates (S1D Fig), in some regions fibrils are present (S1E Fig). Further ^1^H 1D NMR spectra proved to remain reproducibly unchanged over a course of 13 hours. Thus, the following solution condition was finally selected for the H/D exchange experiment: DMSO, 5% D_2_O, and 2% DCA having a pH*-meter read pH of 4.8.

### NMR resonance assignment in the aprotic DMSO solvent

To obtain a residue-specific H/D exchange the individual amide chemical shifts of Aβ(1–42) in the aprotic solvent (i.e. DMSO, 5% D_2_O, and 2% DCA) were assigned by standard backbone triple-resonance spectra recorded (i.e. HNCA, HNCACB, HNCOCA). The sequential backbone walk could be completed to 98% with D1 being the only resonance missing (S2 Fig).

### Quenched H/D exchange experiments

**Fig 1:**
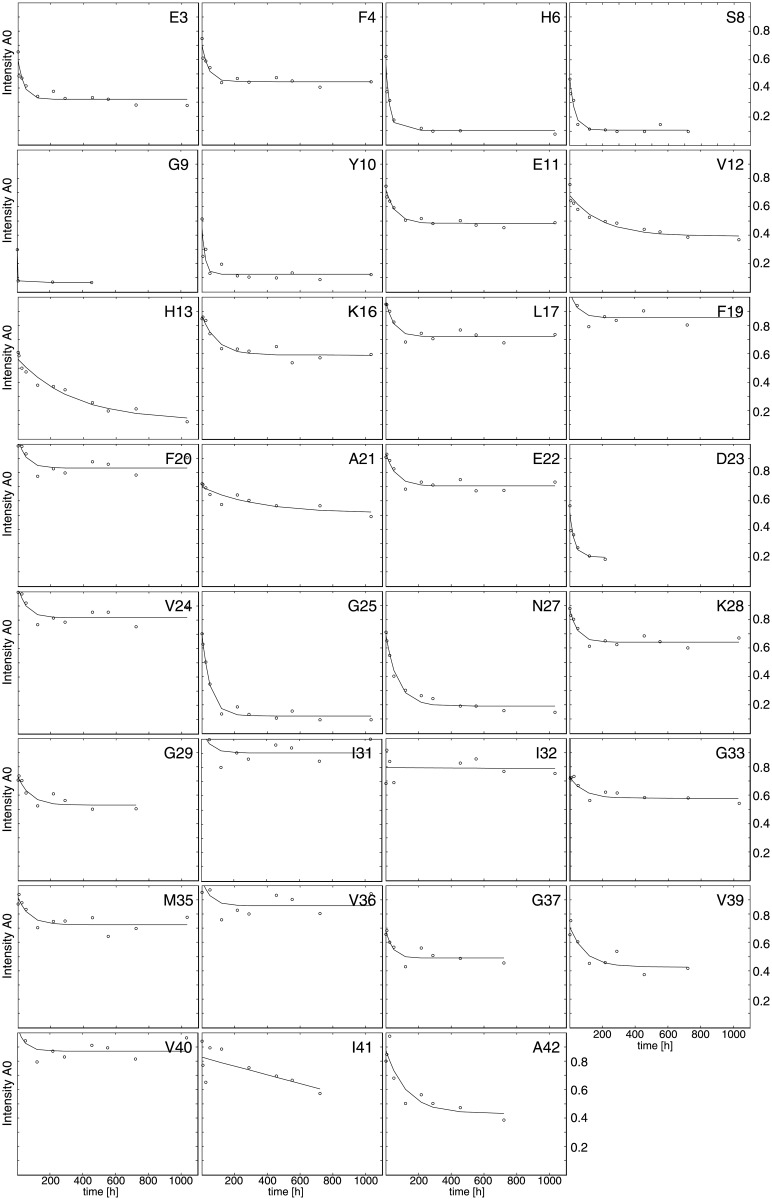
Residue-resolved quenched H/D exchange data for the identification of solvent protected ^15^N-^1^H- moieties of Aβ(1–42) fibrils. The relative peak intensities of the [^15^N,^1^H]-HMQC spectra (y-axis) acquired in the DMSO solution back predicted to time zero are shown for each exchange time in D_2_O as indicated along the x-axis. Smooth solid lines represent the mono- exponential fits of the data points. Some residues (for example: H6, G9, Y10, and G25) show a clear decay towards zero indicative of fast exchange, whereas others (for example F19, F20, I31, and I32) remain at high intensity indicative of a high solvent protection. For many residues the exchange is composed of a fast and a slow exchanging component indicative of the presence of two distinct populations.

Aβ(1–42) was fibrillized at 30 μM at conditions described above, washed with water, and concentrated to approximately 1 mM. The H/D exchange was started by centrifugation of 600 μl of a 1 month old sample for 15 minutes at 178’000 g. Next, the pellet was dissolved in 1.2 ml D_2_O (pD 7) and incubated therein at room temperature between up to 43 days. At a total of 12 different time points within the 43 days a 80 μl aliquot of the sample was taken, centrifuged for 15 minutes at 178’000 g and dissolved into 300 μl of the above mentioned aprotic DMSO solution with a final Aβ(1–42) concentration of approximately 100 μM. Within less than 5 min a 1D ^1^H NMR spectrum followed by 80 time resolved fast-[^15^N,^1^H]-HMQC spectra were measured sequentially for two hours to follow the intrinsic exchange in the aprotic solvent. The intrinsic exchange was fitted individually to back predict the initial intensities over the dead time of the experiment of ca. 5 min for each amide resonance peak that then reflect the number of protons exchanged with deuterium during the incubation time in the fibril sample. For each incubation time point a new sample was prepared as described above and initial intensities had to be referenced to account for slight concentration differences between samples in order to be compared between different incubation time points. These initial intensities were normalized by dividing the amide signal intensities with the peak intensity of the non-exchanging methyl region of the 1D ^1^H NMR spectrum (see Materials and methods). Fig 1 shows the normalized initial intensities versus the incubation time, representing the H/D exchange of amide protons in fibrils over time. For the residues not shown in Fig 1 (i.e. A2, R5, D7, H14, Q15, V18, S26 (S3 Fig), A30, L34 (S3 Fig), and G38) the intrinsic remaining exchange in DMSO or spectral overlap prohibited a detailed analysis.

The inspection of Fig 1 shows immediately that these exchange curves do not follow a single exponential decay, instead they rather represent a biphasic behavior with a fast exchange in the order of hours-days and a very slow exchange close to zero. Such biphasic behaviors were reported for several amyloid systems, e.g. for Aβ(1–42) [16], Aβ(1–40) [18], β2-microglobuline [19], and an SH3 domain [20]. The fast exchange may be due to residual oligomers in the sample or due to molecules at the edge of the fibrils that are in fast exchange with the soluble state, or to local structural heterogeneity [16]. In the following we shall concentrate on the slow exchanges because they are attributed to highly solvent protected and hydrogen-bonded regions of the fibrils. Looking at this part of the data in Fig 1, some residues did not exchange significantly in the time examined (for example F19, F20, I31, and I32), while others moieties show only a relatively fast exchange (for example H6, G9, Y10, G25).

### H/D exchange data

**Fig 2:**
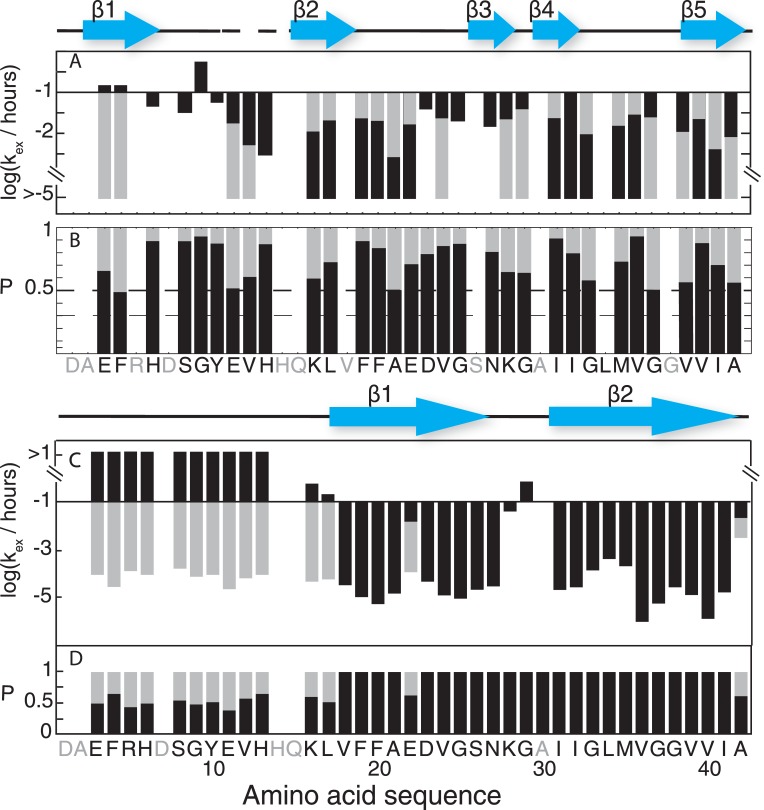
Structural differences of Aβ(1–42) fibrils of this study (A and B) and of Aβ(1–42)^Mox^ fibrils of Lührs et al. (C and D) [16]. They are revealed by the amide exchange rates *k*_ex_ / h^-1^ of individual ^15^N-^1^H moieties and the relative population *P*(F) of the slow and fast exchanging components of the exchange. The exchange rates and population P(F) of Aβ(1–42) fibrils of this study (A and B, colored in grey and black) have been extracted from the H/D exchange curves in Fig 1 and compared with corresponding values of the polymorph studied by Lührs et al. (C and D, colored in grey and black). The blue arrows are indicating the β-strands determined by solid-state NMR of Aβ(1–42) fibrils and the one defined by Lührs et al. and the dashed line indicates missing data. Since the color code follows the main population, following the black colored data in Fig 1A elucidates immediately, that the main population undergoes fast exchange between residues 1–13, slow exchange between residues 16–22, fast exchange between 23–27 (with the exception of V24), slow exchange between 28–36, fast exchange between 37–39 (with no data for G38), slow exchange between 40–41, and fast exchange for residue 42.

Fig 2A shows along the amino acid sequence the exchange data including fast and slow exchange times as well as the relative propensity between the fast and the slow exchanging components. Based on this representation it is evident that in the main population residues 1–13 undergo fast exchange, followed by a slow exchanging segment comprising residues 16–22, and V24 neighbored by fast exchanging residues D23, G25, and N27. From K28-I41 there is again a slow exchanging segment interrupted by the fast exchanging amide moieties of G37 and V39 and accompanied by the less protected C-terminal amide moiety of A42.

Because the slow exchange is for most of the slow exchanging residues much slower than the time span of the experiment only a lower limit of 10^5^ h could be determined (Fig 2A).

## Discussion

### Comparison of the H/D exchange with the solid-state NMR structure of Aβ(1–42) amyloid fibrils

**Fig 3:**
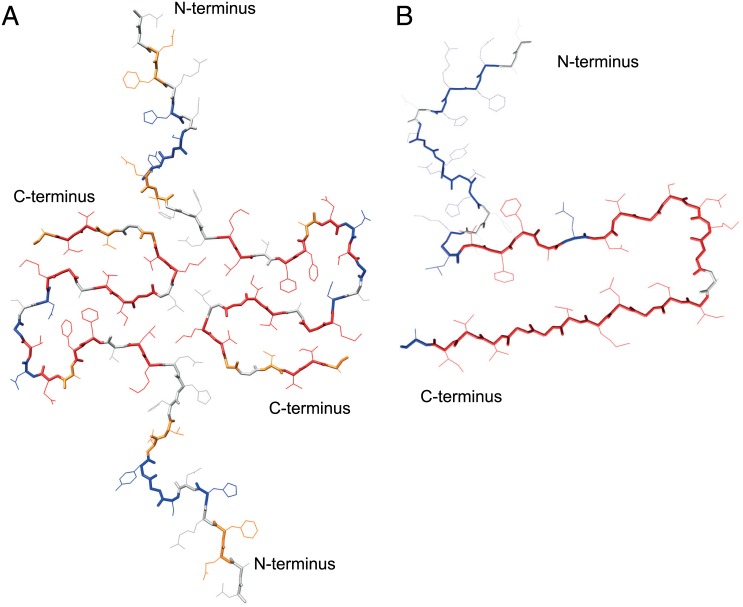
Quenched H/D exchange mapped onto the 3D structure of Aβ(1–42) fibrils. (A) The disease-relevant structure is composed of two molecules per fibril layer [14] (pdb code 2NAO). The double horse shoe-like core structure of the Aβ(1–42) fibrils shows the highest protection. (B) Structure of the polymorph by Lührs et al. [16] (pdb code 2beg). Solvent protected residues are color coded in red, solvent exposed residues in blue, residues with 30–50% protection in orange, and residues lacking data in white.

The quenched H/D exchange analysis is able to reveal insights into the 3D structure of proteins and protein aggregates—in particular the location of secondary structures [12, 13, 16, 21], because hydrogen bonded amide moieties are more protected from the exchange with the solvent compared to the solvent exposed ones. This can readily be seen in the present study by mapping the slow exchanging amide moieties onto the 3D solid-state NMR structure of Aβ(1–42) amyloid fibrils [14, 15] as demonstrated in Figs 2A and 3. The fast exchanging segment comprising residues 1–13 is structurally not well defined in the solid-state NMR structure, while the slow exchanging residues 16–22, 24, and 28–41 are building the hydrophobic double horseshoe core of the fibril structure (Fig 3). Fast exchange is further observed in the loop region connecting β-strand β2 with β3 (i.e. E22), β3 with β4 (i.e. G25, and N27), the loop between β-strand β4 and β5 (i.e. G37 and V39), and at the solvent-exposed C-terminus A42. Interestingly, there is a minor population of slow exchange at the N-terminal amide moieties of residues E3 and F4, which is in line with the secondary chemical shift analysis of the solid-state NMR spectra of Aβ(1–42) fibrils, that indicate an extended well defined minor conformation [14]. Overall, the H/D exchange is in excellent agreement with the solid-state NMR structure of Aβ(1–42) amyloids. This finding allows for a structural comparison between two polymorphs by H/D exchange as we shall discuss next.

### Insights into polymorphism of Aβ(1–42) amyloids by a comparison of the H/D exchange presented with the one published by Lührs et al.

In 2005 Lührs et al. published a quenched H/D exchange of a Aβ(1–42) amyloid fibrils [16]. In contrast to the samples studied here, the Aβ(1–42) of the Lührs et al. study was oxidized at position M35. In addition, the fibrils were grown at pH 7.7 in 10 mM Tris buffer, 150 mM NaCl in contrast to the amyloids studied here that were incubated in 100 mM phosphate with 100 mM NaCl, 100 μM ZnCl, pH 7.4. Based on cryo-electron microscopy it has been suggested that the mass per length of this polymorph was one, while the disease-relevant Aβ(1–42) polymorph here has been determined to have a mass per length of two (meaning the presence of one or two molecules per fibril layer, respectively) [14]. The comparison of the two polymorphs by H/D exchange shown in Fig 2 (Fig 2A versus 2B) further illustrates, that also at the secondary structural level the two polymorphs are distinct. The H/D exchange study of the polymorph by Lührs et al. elucidates a slow exchanging segment comprising residues L17-N27 (interrupted at position E22) followed by the two fast exchanging residues K28 and G29 and a long slow exchanging segment comprising residues I31-A42. The N-terminal segment up to residue K16 shows a bi-exponential decay with a fast as well as a slow exchanging component with similar populations. Based on this H/D exchange two extended β-sheets from L17-N27 and from I31-A42 were suggested. While a similar structural heterogeneity for residues 1–15 is observed in the H/D exchange of the polymorph studied here, the first slow exchanging segment starts already at K16 but extends only to residues D23, a slow exchanging V24 (being part of β-strand β3), followed by fast exchanging residues G25 and N27 and the slow exchanging segment K28-I41 which is interrupted at G37 and V39. Overall, in contrast to the extended slow exchanging segments predicted as two long β-strands of the polymorph of Lührs et al., the polymorph by Wälti et al. shows shorter hydrogen protected regions, which are in agreement with the presence of the four β-strands in the core region of Aβ(1–42) fibrils (Fig 3) [14]. These findings indicate that the two polymorphisms can be classified as segmental polymorphs with different segments forming the cross-β-sheet cores of the two polymorphs [22–24].

In this context it is interesting to note, that the fragments comprising residues 16–21 as well as 35–40 form extended in-parallel β-sheet steric-zipper cross-β structures [24] having thus a structure unlike the corresponding peptide segments in amyloids of full-length Aβ(1–42). Furthermore, the solid-state NMR studies on Aβ(1–40) amyloid fibrils indicate the presence of several polymorphs of segmental-type [3, 25, 26]. Overall, these findings highlight that Aβ may comprise a plethora of distinct amyloid polymorphs.

The presence of a diverse set of polymorphs asks for disease-relevancy. Recent solid state NMR studies on Alzheimer’s disease brain-seeded Aβ amyloids [25] and conformation-specific antibody studies [27] indicate the presence of several polymorphs in brains of Alzheimer’s disease-suffering patients with the Wälti polymorph being one of them [14] and thus disease-relevant, while for the Lührs polymorph only cellular toxicity data is available that indicates the toxicity of the fibrils [16].

In conclusion, the presented quenched H/D exchange of a disease-relevant Aβ(1–42) is in good agreement with the determined solid-state NMR structure and reveals structural differences between two polymorphs.

## Materials and methods

The ^13^C,^15^N-labeled Aβ(1–42) peptide was recombinantly expressed in *Escherichia coli* BL21(DE3) (Sigma Aldrich), and purified as previously published [28]. The fibril sample was also prepared following the protocol published [14, 17]. In short, lyophilized purified Aβ(1–42) peptide was dissolved with 10 mM NaOH with the help of a sonication bath (3 times 30 s sonication with 50–60% power, interrupted by 1 min cooling on ice). To remove large aggregates, the sample was ultracentrifuged for 1 h at 126,000 g. The final sample contained 100 mM sodium phosphate buffer with 100 mM NaCl and 100 μM ZnCl at pH of 7.4. Fibrillization was performed under shaking at 37°C. Seeding was done for 3 generations, 10% of the grand-parent generation was used as seeds for the parent generation, and again 10% for the daughter generation, respectively.

### NMR experiments

For the quenched H/D exchange NMR experiments, a sample of 80 μl of approximately 1 mM ^13^C,^15^N-labeled Aβ(1–42) fibrils were centrifuged for 15 minutes at 178,000 g and dissolved in D_2_O. At 12 time points after 0, 2, 7.5, 24, 50.5, 121.5, 216, 288, 456, 552, 720, and 1032 h an aliquot of fibrils was centrifuged for 15 minutes at 178,000 g and dissolved in DMSO with 5% D_2_O and 2% DCA followed by the immediate measurement of 80 fast-[^15^N,^1^H]-HMQC spectra [29] recorded with 51(*t*_1_) × 1024(*t*_2_) complex points, *t*_1,max_ (^15^N) = 51.3 ms, *t*_2,max_ (^1^H) = 292.5 ms, an interscan delay of 0.2 s, and 4 scans per increment. In addition, 1D ^1^H NMR spectra and EM pictures were recorded to control that most of the fibrils were dissolved and no further dissolution occurred during the time course of the NMR measurements in the DMSO solution.

A total of 960 2D spectra were measured and subsequently analyzed using the script”fit.com” provided by NMRPipe (31). Small variations of the protein concentration between the different time points (probably due to inaccuracies in the preparation) were corrected with the help of the aliphatic region in the 1D ^1^H NMR spectra, which is not affected by the exchange process. Before the measurement of the first point at each time, about 5 min were needed for the dissolution of the fibrils and preparation of the measurement. Therefore, the intensity at time zero had to be back calculated from the measured intensities. Finally, these intensities at time zero of each measurement were plotted versus the exchange time.

For the protein resonance assignment in the DMSO solvent ^13^C,^15^N-labeled Aβ(1–42) was dissolved in DMSO, 2% DCA, and 5% H_2_O and triple resonance experiments were measured on a Bruker 700 MHz spectrometer equipped with a triple resonance cryoprobe at 298 K. Standard pulse sequences were used: 3D HNCA [30] with 100(*t*_1_) × 30(*t*_2_) × 1024(*t*_3_) complex points, *t*_1,max_ (^13^C) = 17.7 ms, *t*_2,max_ (^15^N) = 11.7 ms, and *t*_3,max_ (^1^H) = 90.9 ms, an interscan delay of 0.9 s, and 8 scans per increment; 3D HNCACB [31, 32] with 80(*t*_1_) × 30(*t*_2_) × 1024(*t*_3_) complex points, *t*_1,max_ (^13^C) = 9.1 ms, *t*_2,max_ (^15^N) = 11.7 ms, and *t*_3,max_ (^1^H) = 90.9 ms, an interscan delay of 0.9 s, and 16 scans per increment; 3D HNCOCA (64) with 80(*t*_1_) × 30(*t*_2_) × 1024(*t*_3_) complex points, *t*_1,max_ (^13^C) = 14.2 ms, *t*_2,max_ (^15^N) = 11.7 ms, and a *t*_3,max_ (^1^H) = 90.9 ms, an interscan delay of 0.9 s, and 8 scans per increment. Data were processed with NMRPipe [33] and analyzed with CcpNmr Analysis 2.3 [34].

## Supporting information

S1 Fig Electron micrographs of Aβ(1–42) fibrils. A: Fibrils before dissolution. B: Dissolution in H_2_O. C: Dissolution in DMSO and 5% D_2_O. D and E: Aβ(1–42) fibrils dissolved in a solution containing DMSO, 5% D_2_O, and 0.1% TFA. D represents the major parts of the grid, showing dissolved fibrils and E shows some minor regions containing residual fibrils. F: Aβ(1–42) fibrils dissolved in DMSO, 5% D_2_O, and 2% DCA. In this sample also some residual fibrils were found.
(EPS)

S2 Fig [^15^N,^1^H]-TROSY spectrum of Aβ(1–42) recorded in a DMSO solution at 298 K on a Bruker 700 MHz avance III. The peptide was in a concentration of 91 μM in a DMSO solution with 5% D_2_O and 2% DCA. The cross peaks of the individual ^15^N-^1^H-moieites are labeled with one letter amino acid residue code. The non labeled cross peaks are the N-terminus at 123.5 ppm and the Asn side chains.
(EPS)

S3 Fig Intrinsic H/D exchange of Aβ(1–42) in DMSO. In red the relative peak intensities of the [^15^N,^1^H]-HMQC spectra (y-axis) acquired in the DMSO solution are shown for a few residues indicated for the H/D each exchange time of 121.5 h in D_2_O. In blue are the fit curve to the experimental data shown.
(EPS)
